# Cerebrovascular accidents indicative of COVID-19 infection: about 4 observations in Guinea

**DOI:** 10.11604/pamj.supp.2020.35.2.23751

**Published:** 2020-06-05

**Authors:** Hugues Ghislain Atakla, Kaba Condé, Ayub Neishay, Lounceny Fatoumata Barry, Aissatou Kenda Bah, Mamady Konaté, Mamadou Hady Diallo, Farrah Jasmine Mateen, Fodé Abass Cissé

**Affiliations:** 1 Neurology Department, Ignace Deen University Hospital Center, Conakry, Guinea; 2 Neurosurgery Department, Ignace Deen University Hospital Center, Conakry, Guinea; 3 Rheumatology Department, Ignace Deen University Hospital Center, Conakry, Guinea; 4 Neurology Department, Massachusetts General Hospital, Boston, USA

**Keywords:** Stroke, COVID-19, RT-PCR, Guinea

## Abstract

**Introduction:**

Coronavirus is a virus with potential to target the nervous and respiratory systems. The aim of this work is to establish the prevalence of strokes in COVID19 positive patients in Guinea.

**Methods:**

All patients with stroke confirmed by brain imaging and COVID-positive PCR were included in this study. Retrospective patient data were obtained from medical records. Informed consent was obtained.

**Results:**

The RT-PCR confirmed the initial diagnosis and the chest CT scan provided a good diagnostic orientation. Brain imaging identified ischemic brain lesions. We report the case of four patients with stroke and a COVID-19 incidental finding in Guinea.

**Conclusion:**

This work shows that the onset of ischemic stroke associated with COVID-19 is generally delayed, but can occur both early and late in the course of the disease. More attention is needed because the early symptoms of viral attack are not just pulmonary.

## Introduction

Coronavirus is one of the major viruses that target the human respiratory system, but it also potentially has neuroinvasive capabilities and can spread from the respiratory tract to the central nervous system (CNS) [1]. Neurological complications include stroke, which is a direct consequence of the hypercoagulability associated with COVID-19, and the accompanying sepsis [2,3]. Since the first reports of COVID-19 in December 2019 and its spread to the rest of the world, there have been more than 300,000 deaths to date [4]. According to recent data from a study in Wuhan, China, neurological complications were found in 36% of the 214 patients with SARS-Cov-2, the majority of who were ischemic strokes [4]. However, the disclosure of COVID-19 by stroke is rarely described, and the adequate management of ischemic stroke associated with COVID-19 is not well described. We report in a series of four patients who had a stroke in the context of SARS-CoV-2 infection confirmed by reverse transcriptase PCR (RT-PCR) with the objective of establishing the prevalence of stroke in COVID-19 positive patients in Guinea.

## Methods

We searched the database of the French National Health Safety Agency (ANSS) for patients with acute stroke and suspected characteristics of COVID-19. All patients with image-confirmed, COVID-19 positive strokes were included in the study. Patients who had a complicated inpatient acute stroke pathway or who did not achieve negative RT-PCR or RT-PCR were excluded from the study. Retrospective patient data were obtained from medical records. Informed consent from patients and/or parents was obtained in agreement with the National Health Security Agency (ANSS).

## Results

### CASE NO. 1

**Figure 1:**
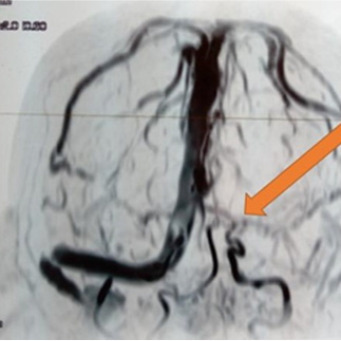
Thrombosis of the right lateral sinus

**Figure 2:**
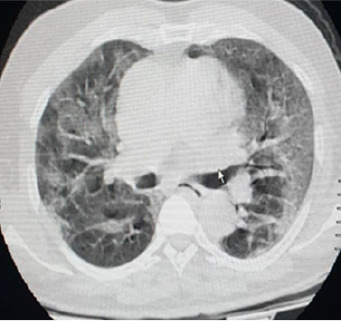
Thoracic CT showed peripheral involvement with diffuse frosted glass opacities

It was a 71-year-old man admitted to the emergency room of the Ignace Deen National Hospital for left hemi-body weakness, headache, and partial right hemi-body seizure with a fever of 38.6°. His history included high blood pressure, dyslipidemia. There was no known of COVID- 19 exposure. At the anamnesis, the patient had an altered mental state and non-icteric with a blood pressure of 140/80 mm Hg, a tachycardia of 109 beats per minute, an oxygen saturation of 88%. Neurological examination revealed a pyramidal syndrome of the left flasco-spastic hemi-body, rated at 2/5 proportional. The rest of the examination was unremarkable. MRI and cerebral MRI showed multiple cerebral infarctions with right lateral venus sinus thrombosis visible on the MRI (Figure 1). A curative dose of anticoagulation was initiated. Eighteen hours later, the patient was transferred to the intensive care unit for obnubilation of consciousness with a Glasgow score of 12/15; with dyspnea and a fever of 39°. The neurological examination was stationary, the cardiopulmonary examination showed tachycardia and tachypnea at 30 beats/minute with bilateral rales at the bases. The biologic workup showed lymphopenia. A biological inflammatory syndrome with a CRP of 38 mg/dl. The D-dimer was 90000μg/L. Thoracic CT scan showed peripheral involvement with diffuse frosted glass opacities (Figure 2). The COVID-19-PCR test was positive. Thus the clinical, imaging results and the COVID-PCR test positive the diagnosis of stroke revealing COVID-19 was retained. The patient was started on hydroxychloroquine, Azithromycin and oxygen therapy. After 48 hours in isolation, the patient died in a cardiopulmonary arrest.

### CASE NO. 2

**Figure 3:**
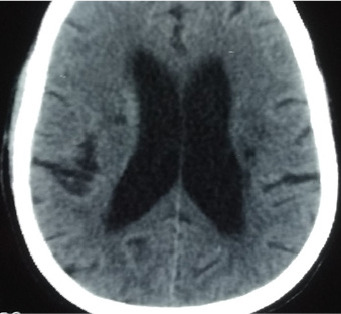
Multiple lacunar infarction

**Figure 4:**
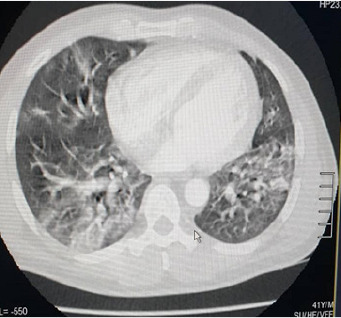
Uneven airspace opacities and frosted glass diffuse opacities characteristic of COVID-19 viral infection and pulmonary embolism

The case involved a 58-year-old man with no significant medical history, who presented for sudden onset of left hemiparesis, acute mental confusion, physical asthenia with fever and no known contact with a patient declared positive for COVID-19 according to his entourage. On examination there was tachycardia at 135 beats/minute, tachypnea at 28 cycles/minute, oxygen saturation at 85% and fever at 40°C. The patient was put on oxygen. The emergency ECG showed a complete arrhythmia with atrial fibrillation and a disorder of repolarization. Brain CT scan revealed multiple lacunar infarctions (Figure 3). Within 24 hours of hospitalisation, the patient developed permanent respiratory dyspnea with shivering. The D-dimer was 2450 μg/L. Thoracic CT scan showed peripheral involvement with uneven airspace opacity and diffuse frosted glass opacity, suggestive of COVID-19 viral infection and pulmonary embolism (Figure 4). RT-PCR confirmed a SARS-CoV-2 infection and he was treated with a curative dose anticoagulant, carvedilol 6.25mg, azithromycin 500mg. The patient was transferred to the Centre for Infectious Disease Control for follow-up for SARS-CoV-2. With a recommendation for therapeutic abstention from hydroxychloroquine. After 16 days under observation, including 4 consecutive days without clinical symptoms related to COVID-19, and RT-PCR negative on 2 occasions. The patient was declared cured with COVID-19. He was discharged and transferred to the Neurology Department for neurological sequelae.

### CASE NO. 3

**Figure 5:**
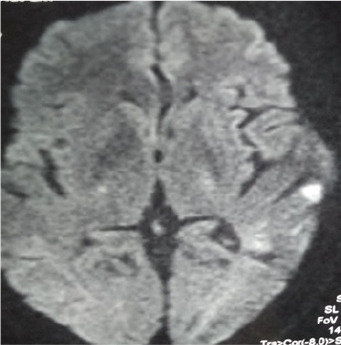
Bi-hemispheric multiple hypersignals suggesting multiple cerebral infarctions

**Figure 6:**
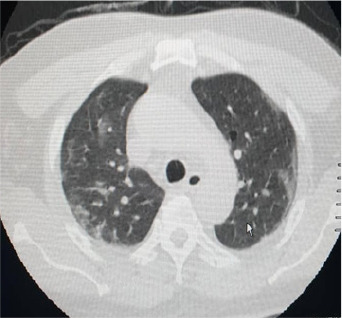
Bilateral diffused frosted glass diffused infiltrates

The patient was a 62-year-old woman with a history of hepatitis B, hypertension, type II diabetes and dyslipidemia. She was admitted to the emergency room for right hemi-body weakness, dysarthria, and recent hyposmia in a febrile context with the known exposure to a confirmed case with COVID-19, although this was discovered after admission. General examination revealed tachycardia at 103 beats per minute, blood pressure at 170/90vmm Hg, and temperature at 37.5°C. Cerebral MRI showed bi-hemispheric multiple hypersignals suggestive of multiple cerebral infarctions (Figure 5). The D- dimer was 11847 μg/L. Three days after admission, the patient developed tachypnea-like respiratory symptoms with progressive oxygen desaturation. RT-PCR confirmed a SARS-CoV-2 infection and chest CT scan revealed bilateral diffuse frosted glass infiltrates (Figure 6). The patient was isolated and treated with low molecular weight heparin, an antiplatelet aggregation (acetylsalicylic acid 160mg); hydroxychloroquine 300mg and azithromycin 500mg. She was declared cured 21 days later following the absence of clinical and biological symptoms related to VCOS-RAS-2. The patient was discharged and referred for physical therapy for motor rehabilitation.

### CASE NO. 4

The patient was a 53-year-old hypertensive male with type II diabetes who was admitted to the emergency department with transient 25- to 30-minute episodes of right hemiparesis and motor aphasia, hyposmia and recent agueusia with severe physical asthenia. The case history revealed a known exposure with one asymptomatic confirmed case of COVID-19. Vital signs were within normal limits with a fever of 38°C. Initial neurological examination was normal. The brain scan performed did not reveal any acute brain injury. Thus the diagnosis of transient ischemic attack was made. RT-PCR confirmed coronavirus infection and the D-Dimer was 723 μg/L. The chest CT scan was normal. The patient was treated with low molecular weight heparin, hydroxychloroquine 300mg; aspirin, statin, and was transferred to the Infectious Disease Control Centre for follow-up for SARS-COV-2. Following 2 negative RT-PCR 48 hours apart, the patient was declared cured and released (Table 1).

**Table 1:** Patient distribution by clinical and Para clinical observations

Observations	Patient 1	Patient 2	Patient 3	Patient 4	Total n (%)
Clinical/Para clinical					
Age (years)	75	58	62	53	
Metabolic Syndrome	+	+	+	+	4 (100%)
Notion of patient contact COVID-19	-	-	+	+	2 (50%)
Motor deficit; tachycardia; hypoxemia	+	+	+	+	4 (100%)
Disturbance in alertness	+	+	-	-	2 (50%)
Hyposmia	-	-	+	+	2 (50%)
Agueusia	-	-	-	+	1 (25%)
Fever	+	+	+	+	4 (100%)
D-Dimer ˃ 1000	+	+	+	-	3 (75%)
Thoracic CT (frosted glass infiltrates)	+	+	+	+	4 (100%)
Cerebral CT (Multiple Cerebral Infarction)	+	+	+	-	3 (100%)
Cerebral CT (Multiple Cerebral Infarction + Cerebral Venous Thrombosis)	+	-	-	-	1 (25%)
Normal Brain CT	-	-	-	+	1 (25%)
Death	+	-	-	-	1 (25%)
Healed	-	+	+	+	3 (75%)

Abbreviation: (n) = Effectif; (%) = Percentage; (+) = Yes; (-) = No

## Discussion

Although ischemic stroke has been recognized as a serious complication of COVID-19 [3]. Seventy-three (73) days after the announcement of the first case confirmed positive to COVID-19 in Guinea, there were 3176 cases of which four (4) cases were revealed by a cerebrovascular accident, a frequency of 0.12%. The ischemic strokes at the site of COVID-19 infection are probably due to the sepsis and hypercoagulability associated with COVID-19, which may predispose to stroke [3]. Hypertension; dyslipidemia and sedentariness were the most common comorbidities found in ¾ of our patients, i.e. 75%. Fei Zhou and colleagues in Wuhan, China, in a study conducted on 191 patients, reported 91 (48%) patients with comorbidities [5]. Hypertension was the most common (58 patients (30%)), followed by 36 diabetic patients (19%); and coronary heart disease in 15 patients (8%) [5]. These pre-existing medical conditions would precipitate thrombus formation and stroke-causing emboli in COVID-19 positive patients. All patients had focal neurological deficit; hypoxia; fever above 38°C with compensatory tachycardia while 2(50%) reported hyposmia versus 25% agueusia.

All our patients were asymptomatic at COVID-19 before the vascular event occurred. This result is comparable to that reported by Thomas J. Oxley in a series of 5 cases carried out in New York which found 3 asymptomatic cases at COVID-19 before the stroke and 2 symptomatic cases before the stroke [6]. These results suggest that the ischemic stroke associated with COVID-19 is generally delayed, but may occur both early and late in the disease course. However, these data are nonetheless limited to confirm such a suggestion. D-Dimer was greater than 500 μg/L in all our patients with a low representation in patient N°4. This seems to justify the absence of respiratory symptoms and transient neurological disorders in this patient, although this observation deserves further reflection on a broader set. In addition, elevated D-Dimer levels in the COVID-19 field may indicate an elevated inflammatory state and coagulation cascade abnormalities that may play a role in stroke [7]. Viral infections are known to cause stroke by increasing the risk of embolism [8]. Lumbar puncture was not performed in this study however, there are already reports of SARS-CoV-2 identified in cerebrospinal fluid by PCR [9]. Chest CT and brain imaging revealed frosted glass infiltrates and multiple cerebral infarctions, respectively, in all our patients with associated cerebral venous thrombosis. Our observation is consistent with that of Beirut R, which describes identical thoracic lesions and thromboembolic cerebral lesions in a series of 6 patients [10].

Now the question of management of patients suffering from ischemic stroke related to COVID-19 infection in a highly prothrombotic context becomes inherent to our function given the lack of therapeutic evidence and the major risks associated with their use [11,12]. We currently have no protocol for the management of these patients. Heparin therapy has been systematically used in this work in order to limit the repercussions of the prothrombic state on vessels and brain tissue. Hydroxychloroquine was not administered in patients with significant cardiovascular risk factors. For the cardiac arrhythmia-like side effects of the molecule in the context of a virus-related thromboembolic cascade would be a vicious circle and would even increase the risk of stroke occurrence and recurrence. The rest of the management has been symptom-oriented. The evolution was marked by the death of case N°1 and the recovery of the three other patients after an average of 3 weeks of observation. Other studies are underway to better characterize Neurocovid in Guinea.

## Conclusion

Our observations suggest that further research is needed to identify the neurological implications of COVID-19 disease given the occurrence of central and peripheral neurological manifestations. Plans should be developed to ensure that the management of stroke is not neglected, although the control of COVID-19 infection is our highest priority. We believe that autopsies of the brain will lead to a better understanding of the mechanisms involving the CNS in the pathophysiology of Neurocovid and thus facilitate management. Ultimately, primary prevention of infection and stroke seems to be the best option available to us.

### What is known about this topic

- The aggression of the coronavirus on the body leads to neurovascular complications.

### What this study adds

- Stroke can also be indicative of COVID-19 infection.

## Competing interests

The authors declare no competing interests.
